# Mechanical and Biological Advantages of a Tri-Oval Implant Design

**DOI:** 10.3390/jcm8040427

**Published:** 2019-03-28

**Authors:** Xing Yin, Jingtao Li, Waldemar Hoffmann, Angelines Gasser, John B. Brunski, Jill A. Helms

**Affiliations:** 1 State Key Laboratory of Oral Diseases & National Clinical Research Center for Oral Diseases, West China Hospital of Stomatology, Sichuan University, Chengdu 610041, China; yinxing@scu.edu.cn (X.Y.); lijingtao86@163.com (J.L.); 2 Division of Plastic and Reconstructive Surgery, Department of Surgery, Stanford School of Medicine, Stanford, CA 94305, USA; brunsj6@stanford.edu; 3 Nobel Biocare Services AG, P.O. Box, Zurich-Airport, 8058 Zurich, Switzerland; waldemar.hoffmann@nobelbiocare.com (W.H.); angelines.gasser@nobelbiocare.com (A.G.)

**Keywords:** dental implant, osseointegration, alveolar bone remodeling/regeneration, bone biology, finite element analysis (FEA), biomechanics

## Abstract

Of all geometric shapes, a tri-oval one may be the strongest because of its capacity to bear large loads with neither rotation nor deformation. Here, we modified the external shape of a dental implant from circular to tri-oval, aiming to create a combination of high strain and low strain peri-implant environment that would ensure both primary implant stability and rapid osseointegration, respectively. Using in vivo mouse models, we tested the effects of this geometric alteration on implant survival and osseointegration over time. The maxima regions of tri-oval implants provided superior primary stability without increasing insertion torque. The minima regions of tri-oval implants presented low compressive strain and significantly less osteocyte apoptosis, which led to minimal bone resorption compared to the round implants. The rate of new bone accrual was also faster around the tri-oval implants. We further subjected both round and tri-oval implants to occlusal loading immediately after placement. In contrast to the round implants that exhibited a significant dip in stability that eventually led to their failure, the tri-oval implants maintained their stability throughout the osseointegration period. Collectively, these multiscale biomechanical analyses demonstrated the superior in vivo performance of the tri-oval implant design.

## 1. Introduction

Implants have undergone a nearly continual transformation since their inception. Variations in fabrication materials, surface texture, coating, and taper have yielded implants that osseointegrated and are clinically successful [1,2,3,4,5]. Most dental implants, however, still have a circular cross-section, which reflects their origins as titanium screws [6,7].

A non-circular cross-section may have advantages. When placed into a cylindrical osteotomy, conventional implants typically have a uniform bone-implant contact (BIC), and the resulting peri-implant strains are uniformly distributed around its circumference [8]. Although the relationship is not straightforward [9], it is generally presumed that the greater the amount of bone-implant contact (BIC) the better is implant stability [10].

A non-circular, e.g., a tri-oval shaped implant, on the other hand, would be predicted to engage bone on its vertices, or tri-oval maxima, which would provide mechanical stability and result in peri-implant strains concentrated at these regions.

Depending on the extent of tri-ovality, there would also be sites of minimal BIC. An extensive literature has shown that new woven bone first forms in areas where BIC is absent [8,11,12,13,14].

In previous studies, we demonstrated that when an implant is placed with high insertion torque (IT), then peri-implant bone is compressed and osteocytes within this bone begin to die [8,15,16].

Some proposed embodiments of dental implants have had non-circular cross-sectional shapes to reduce “friction between the bone and implant during insertion” [17,18]. Once the implant is in place, however, it is not friction but rather peri-implant stresses and strains that appear to be most important: Inserting an implant creates strains in peri-implant tissues [11,19,20], and the magnitude of these strains has a direct, quantifiable impact on the behavior of cells and tissues in the peri-implant environment [20,21].

In areas where an implant contacts bone, the stiff interface stabilizes the implant [15]. There is a biological downside to this relationship, though: if the implant is placed with high IT, then the stiff interfacial bone is compressed to a greater extent, and the result is higher strain. Cells within the bone matrix, i.e., osteocytes, respond to this high strain by dying [8,15,16].

The converse is also true: in areas of low strain, fewer peri-implant osteocytes die and bone resorption is minimal [22]. If the peri-implant bone is “soft”, e.g., has a trabecular microstructure, then cells in the low strain environment tend to proliferate. Ultimately, these cells can differentiate into osteoblasts and osseointegration ensues [22].

Once osteocytes have died, necrotic bone is resorbed via an osteoclast-mediated process [8,15]. Thereafter, new bone formation ensues [8,23]. The resorption of dead peri-implant bone, however, jeopardizes implant stability. We speculated that there could be a way to avoid this by purposefully creating a combination of high strain and low strain peri-implant environments that would ensure both mechanical engagement in the surrounding bone, i.e., primary stability, and rapid osseointegration, respectively. In a tri-oval implant design, the maxima regions would theoretically correspond to areas of higher strain and provide initial mechanical stability. The minima regions of the tri-oval design would theoretically correspond to areas of low strain and constitute pro-osteogenic zones where new bone formation would contribute to secondary implant stability. Here, we tested the veracity of this theory by comparing outcomes of tri-oval and round implants placed into healed maxillary sites according to a well-established in vivo mouse model of oral implant osseointegration.

## 2. Materials and methods

### 2.1. Implant Design

Implants were manufactured from CP Titanium Grade 4 with a TiUnite surface (Nobel Biocare AB, Goteborg, Sweden). Geometries for round (control) and tri-oval implants are described in Supplemental Table S1.

### 2.2. Animals and Tooth Extraction Surgeries

Procedures were approved by Stanford Committee on Animal Research (protocol #13146) and conformed to the ARRIVE guidelines. Wild-type C57BL/6 mice (Jackson Laboratory, Bar Harbor, ME, USA, #003291) were housed in a temperature-controlled environment with 12h light/dark cycles. In total, 96 eight-week-old male mice were used.

### 2.3. Implant Placement, Osteotomy Site Preparation, and Experimental Groups

Extraction of bilateral maxillary 1st molars (mxM1) was performed using forceps. Bleeding was controlled by local pressure. Extraction sockets were allowed to fully heal for four weeks [22]. Pain control was ensured by daily delivery of analgesics. Immediately following surgery mice received sub-cutaneous injections of buprenorphine (0.05–0.1 mg/kg) for analgesia once a day for a total of three days. Animals were fed with regular hard chow diet. Daily monitoring revealed no evidence of prolonged inflammation during healing at the surgical sites. No antibiotics were given to the operated animals.

Following anesthesia, osteotomy sites were produced using a dental engine (NSK, Tokyo, Japan) and a 0.45mm diameter drill bit at 800 rpm (Drill Bit City, Prospect Heights, IL, USA). Aseptic saline was used for irrigation during the drilling process.

A split-mouth design was employed for this study, wherein each mouse received one round implant and one tri-oval implant. See Supplemental Table S2 for the distribution of groups and analyses performed in each group. Implants were placed either below the occlusal plane or at the level of occlusion.

### 2.4. Implant Insertion Torque Measurement

To compare the insertion torque (IT) of the round and tri-oval implants, two independent experimental setting were performed. In one experimental design, holes (0.45mm diameter) were produced in a uniform block of poplar wood and then round and tri-oval implants were inserted all the way into the wood block. The IT was then recorded by attaching the implants to a miniature torque cell (MRT Miniature Flange Style Reaction Torque Transducer, Interface Inc., Scottsdale, AZ, USA). Poplar wood had an elastic modulus of 10.9 GPa [24], which is on the same order of magnitude as dense bone (e.g., 10–20 GPa) [25].

In another experimental design, the IT was measured directly on mice [26]. Osteotomies (0.45mm in diameter) were prepared in the healed maxillary tooth extraction sites and the implants were inserted. The animals were sacrificed immediately after implant placement. The mandible was removed to fully expose the inserted implant, and the IT was then measured by connecting the implant to a pre-calibrated hand-held gauge (Tonichi, Tokyo, Japan).

The rationale for comparing insertion torques (IT) of round and trioval implants in wood was not to imply that wood is an excellent substitute test material for bone; rather it was because (a) wood offered a uniform material allowing side-by-side IT tests of round and trioval implants under identical conditions, and (b) the IT tests in wood could be conducted using a sensitive miniature torque transducer that could not be used in vivo.

### 2.5. Lateral Stability Testing, Finite Element Modeling, and Calculation of Elastic Modulus

A lateral stiffness test (LST) of implants in alveolar bone was carried out using maxillae samples retrieved on PID 0, 3, 7, 14, and 20. The LST was based on an assumed linear relationship between a lateral force exerted on the top of an implant and the resulting lateral displacement of the implant in bone. Our experience with this method, including modeling with finite element analysis indicates that this assumption is valid for displacements in the range of about 0 to 50 µm [15,27].

**Figure 1:**
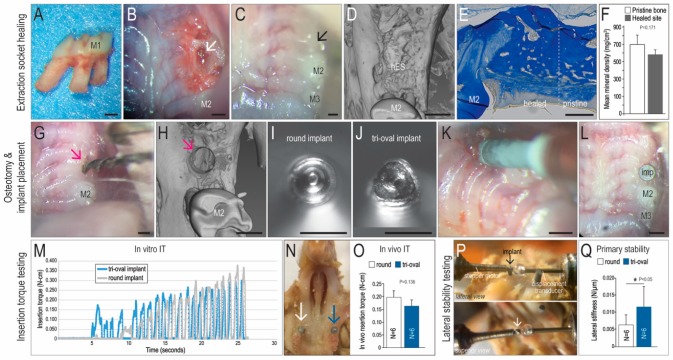
Tri-oval implants placed in type III bone with the same insertion torque exhibit higher primary stability as compared to conventional round implants. (**A**) Maxillary first molars (M1) were extracted from skeletally mature (8-week-old) male mice. (**B**) Intraoral photos of extraction socket (white arrow) and (**C**) Healed extraction site (black arrow). (**D**) Representative micro-CT imaging and (**E**) Representative aniline blue staining of the healed extraction socket on PED28. (**F**) Quantification of mean bone mineral density (BMD) on PED28, where the BMD of the healed extraction site was equivalent to surrounding pristine alveolar bone. (**G**) Osteotomies (0.45 mm dia.; pink arrow) were produced in the healed extraction sites using dental drill. (**H**) Representative micro-CT image of the prepared osteotomy site. (**I**) Geometries of the round and (**J**) tri-oval implants in cross-section. (**K**) Implant placement surgery. (**L**) Implants were positioned at the height of the gingiva. (**M**) In vitro IT testing and (**N**) In vivo IT testing where the white arrow indicates a round implant; blue arrow indicates a tri-oval implant. (**O**) Quantification of in vivo IT for round (white) and tri-oval (blue) implants. (**P**) Lateral stability testing of round and tri-oval implants (arrows) in the mouse maxillae; a stepper motor laterally displaces the implant a known amount while the force to do so is measured by a transducer. (**Q**) Tri-oval implants are significantly more stable than round implants at the time of insertion. Abbreviations: M1, maxillary first molar; M2, maxillary second molar; M3, maxillary third molar; hES, healed extraction site; PED, post-extraction day; imp, implant; IT, insertion torque. Scale bars = 500 µm.

To carry out LST, the animals were sacrificed and the skulls, with the maxillae removed and sectioned in half sagittally, were submerged in 100% ethanol. The half-maxilla containing the implants was then rigidly clamped to a solid support so that the implant was positioned between a linear actuator (Ultra Motion Digit D-A.083-AB-HT17075-2-K-B/3, Mattituck, NY, USA) equipped with an in-line 10 N force transducer (Honeywell Model 31), and a displacement transducer (MG-DVRT-3, Lord MicroStrain, Williston, VT, USA). A tare load of 0.05 N was applied to one side of the implant while the stylus of the displacement transducer was positioned against the diametrically-opposite side of the implant. Under software command, the actuator was triggered to deliver three cycles of a displacement vs. time waveform with a peak displacement of about 30 µm (Figure 1M). The force was applied, and the resulting lateral displacement of the implant was measured at a consistent height of ~0.5 mm above the crest of the maxillary bone. Previous tests and calculations show that under the force conditions in this test, there is negligible deformation of the titanium implant, meaning that virtually all lateral displacement arises from displacements in the peri-implant tissue. Lateral force and lateral displacement of the implant were recorded and stored to disc for later data analysis and calculation of the ratio between force and displacement, i.e., lateral stiffness (in Newtons/micron).

Finite element (FE) modeling provided insight into the relationship between the experimentally-measured lateral stiffness and the elastic properties of the surrounding peri-implant bone [28]. Based on stiffness values from lateral stability testing at post-implant day 3 (PID3), a FE model was used to estimate the elastic modulus of peri-implant tissue. A computer-aided design (CAD) file of each implant was obtained from the manufacturer (Nobel Biocare AB, Göteborg, Sweden) and imported into COMSOL Multiphysics 5.3 when formulating models of the lateral stiffness testing (LST). Each implant was installed to full depth (i.e., eight threads) into a 0.45 mm drill hole made completely through a cylinder (2 mm diameter, 1.45 mm height) of uniform bone having a Young’s elastic modulus and Poisson’s ratio selected so that the lateral stiffness computed from the FE model matched the experimentally-measured lateral stiffness. A no-slip boundary condition was applied between implant and bone, and the side and bottom surfaces of the bone cylinder were fixed in space. In the FE model simulating LST, a 0.2N lateral load was applied on the side of the implant’s top portion, at a height of 0.58 mm above the surface of the bone. The direction of the applied force was perpendicular to the long axis of the implant. The resulting displacement of the implant in the same direction of the lateral force was measured from the displacement output. The ratio of the applied lateral force to the measured lateral displacement at 0.58 mm above the surface of the bone was defined as the lateral stiffness. A typical FE model formulated as described above involved about 238,000 degrees of freedom. To match the results from a given experimental stiffness test of a round or tri-oval implant, the Young’s elastic modulus of the bone in the model was parametrically changed until there was a match in lateral stiffness between the FE model and the actual experiment. These FE models demonstrated that the lateral stiffness strongly depended on the Young’s elastic modulus of the peri-implant bone.

### 2.6. Calculating Elastic Modulus of Peri-Implant Bone as a Function of Lateral Stability

Implant insertion caused dynamic tissue remodeling, which could potentially change the tissue elastic modulus in the peri-implant region. Although the changes in peri-implant elastic modulus could not be measured directly on mice, we used FE modeling to generate estimates basing on stiffness values from lateral stability testing at PID3. In the round implant cases, the mean lateral stiffness was 0.00198 N/µm, which corresponded to a modulus of ~2.6 MPa for the peri-implant bone. In the tri-oval implant cases, the mean lateral stiffness was 0.00689 N/µm, which corresponded to a modulus of ~9.2 MPa, a 3.5 times stiffer peri-implant bone than in the case of the round implants.

### 2.7. Sample Preparation, Tissue Processing, and Histology

Mice were euthanized on PID 3, 7, 10, 14, and 20. For those animals whose implants were to be subjected to mechanical testing, maxillae were harvested with skin and superficial muscles removed, fixed in 100% ethanol, and then subjected to lateral stiffness testing. In cases where implants were evaluated by histology/histomorphometry, tissues were fixed in 4% paraformaldehyde overnight at 4 °C then decalcified in 19% EDTA.

After complete demineralization, specimens were dehydrated through an ascending ethanol series and underwent clearing in xylene prior to paraffin embedding. Before immersion in xylene, implants were gently removed from the samples. Eight-micron-thick longitudinal sections were cut and collected on Superfrost-plus slides [27]. Tissue sections prepared for histology, immunohistochemistry, and immunofluorescence were prepared by one individual then quantified by a blinded individual.

Aniline blue staining was performed to detect osteoid matrix. Tissues sections were also stained with the acidic dye, picrosirius red, to discriminate tightly packed and aligned collagen molecules. Viewed under polarized light, well-aligned fibrillary collagen molecules present polarization colors of longer wavelengths (red) as compared to less organized collagen fibrils that show colors of shorter (green-yellow) wavelengths [27].

### 2.8. Histomorphometry

Maxillae were embedded in paraffin and sectioned in the transverse planes. The space occupied by the 0.5mm implant was represented across ~60 tissue sections, each of which were 8 µm thick. Of those 60 sections, a minimum of four Aniline blue-stained tissue sections were used for the quantification of new peri-implant bone formation. Each section was photographed using a Leica digital imaging system at 5× and 10× magnification. The digital images were analyzed using ImageJ software 1.4 (National Institute of Mental Health, Bethesda, MD, USA). The percentage of aniline blue-positive new bone (%NB) was calculated using the area occupied by aniline-blue-positive pixels divided by the total number of pixels in the defined region of interest (ROI). Pixel counts from these individual tissue sections were performed in triplicate then averaged for each sample.

### 2.9. TUNEL Staining, Alkaline Phosphatase Activity, and Tartrate Resistant Acid Phosphatase Activity

TUNEL staining was performed as described by the manufacturer. Briefly, sections were incubated in proteinase K buffer (20 μg/mL in 10 mM Tris pH 7.5), applied to a TUNEL reaction mixture (In Situ Cell Death Detection Kit, Roche, Mannheim, Germany), and mounted with DAPI mounting medium (Vector Laboratories, Burlingame, CA, USA). Slides were viewed under an epifluorescence microscope.

Alkaline phosphatase (ALP) activity was detected by incubation in nitro blue tetrazolium chloride (NBT; Roche, Mannheim, Germany), 5-bromo-4-chloro-3-indolyl phosphate (BCIP; Roche, Mannheim, Germany), and NTM buffer (100 mM NaCl, 100 mM Tris pH 9.5, 5 mM MgCl). After its development, the slides were dehydrated in a series of ethanol and xylene and subsequently cover-slipped with Permount mounting media (Thermo Fisher Scientific, Waltham, MA, USA).

Tartrate-resistant acid phosphatase (TRAP) activity was observed using a leukocyte acid phosphatase staining kit (Sigma, St. Louis, MO, USA). After its development, the slides were dehydrated in a series of ethanol and xylene and subsequently cover-slipped with Permount mounting media (Thermo Fisher Scientific, Waltham, MA, USA).

### 2.10. Micro-CT Imaging

Scanning and analyses followed published guidelines [29]. Ex vivo high-resolution acquisitions (VivaCT 40, Scanco, Brüttisellen, Switzerland) at 10.5 μm voxel size (55 kV, 145 μA, 347 ms integration time), were performed on post-extraction days 28 and immediately after drill preparation. Multiplanar reconstruction and volume rendering were carried out using OsiriX software (version 5.8, Pixmeo, Bernex, Switzerland).

### 2.11. Statistical Analyses

For lateral stiffness tests, results are presented as the mean ± 95% confidence interval. In testing for differences among five means in the stiffness tests for the round or the tri-oval implants at PID 0 through 20, we used one-way ANOVA with PID time as the factor. In comparing the stiffness of round vs. tri-oval implants at any given time point (PID), Student’s t-test was used to quantify differences. *p* ≤ 0.05 was significant.

## 3. Results

### 3.1. Tri-oval Implants Exhibit Higher Primary Stability Compared to Round Implants

Most dental implants are placed into healed sites [30]; to recapitulate this clinical condition, maxillary first molars (mxM1) were extracted from skeletally mature mice (Figure 1A,B). Within seven days, soft tissue healing was complete (Figure 1C). After four weeks, sites were evaluated clinically, by µCT imaging (Figure 1D), and by histology (Figure 1E), which together confirmed complete healing (Figure 1F).

A split-mouth design was then used: osteotomies were produced in healed sites (Figure 1G,H) and two implants were placed, one round (Figure 1I) and the other tri-oval (Figure 1J). All implants were placed ~0.5 mm above the alveolar bone crest and below the plane of occlusion (Figure 1K,L). Insertion torque (IT) was measured using in vitro and in vivo methods. Both analyses indicated that IT values were equivalent between the round and tri-oval implants (Figure 1M,N,O). Primary stability was measured (Figure 1P) and these lateral stability tests demonstrated that tri-oval implants had significantly higher primary stability than round implants (Figure 1Q). How was this greater primary stability achieved?

### 3.2. The Maxima of a Tri-Oval Implant Provide Higher Stability

**Figure 2:**
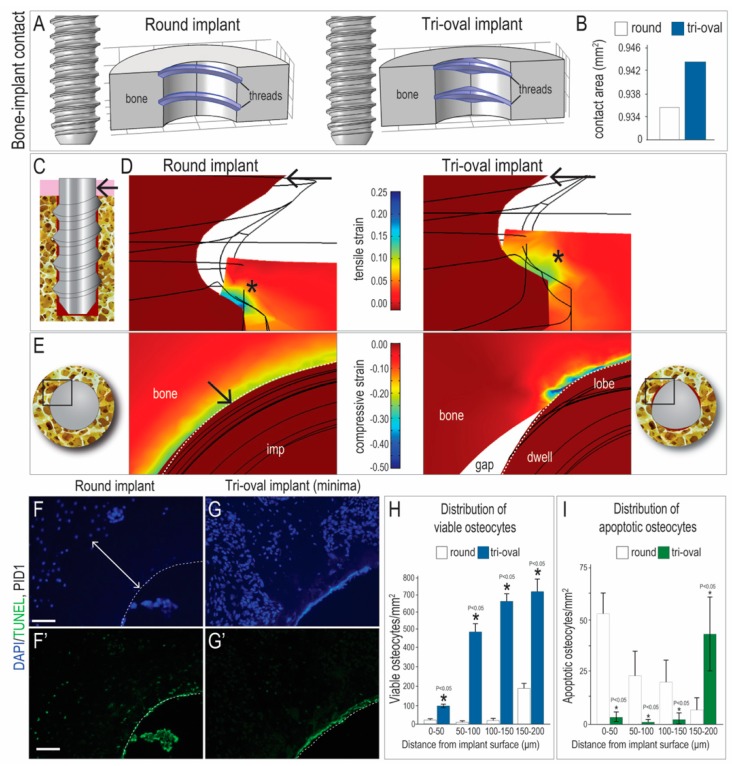
Compared to a round implant, the minima of a tri-oval implant are associated with significantly lower strains and a significantly smaller zone of osteocyte death. (**A**) FE modeling of round (left) and tri-oval (right) implants in bone, using CAD files of the actual implants used in vivo. In a transverse plane, the threads of each type of implant (blue) penetrate the bone, which is modeled as a solid material. (**B**) The calculated bone-implant contact area due to thread penetration. (**C**) Formulation of a FE model of laterally-loaded implant in bone. (**D**) Peri-implant strains surrounding laterally-loaded round and tri-oval implants in the sagittal plane. (**E**) Peri-implant strains arising from initial misfit of the round and tri-oval implants as seen in the transverse plane; only the maxima of the tri-oval implant penetrate the bone. (**F**) DAPI staining of interfacial bone surrounding a representative round implant and (**G**) a representative tri-oval implant; white arrow denotes a circumferential osteocyte-free zone and dotted white line demarcates the osteotomy edge. (**F’**, **G’**) TUNEL staining on adjacent tissue sections. Quantification of the distribution of (**H**) viable and (**I**) apoptotic osteocytes as a function of distance from implant. Abbreviations: imp, implant; PID, post-implant day. Scale bars = 50 µm.

Computational models were generated to determine whether a difference in contributed to the higher primary stability of tri-oval implants. These analyses showed that the threads of a round implant penetrated ~25 µm into bone whereas for a tri-oval implant, the maxima penetrated ~45 µm into bone (Figure 2A). Despite the fact that minima regions were not in contact with bone, a tri-oval implant still had a larger calculated BIC (Figure 2B).

We used FE modeling to understand how the difference in BIC affected peri-implant strains and, in turn, lateral stiffness of the implants. Lateral loading was simulated in the FE model (arrow, Figure 2C) and in both cases the resulting strains concentrated at sites of BIC (Asterix, Figure 2D). The magnitude of these strains, however, was higher in the round implant case (Figure 2D). This meant that when exposed to the same lateral force, the stability of the tri-oval implant was greater than that for the round implant.

The distribution of the peri-implant strains was different, depending on the implant geometry. For example, the round implants had a circumferential zone of high strain whereas the tri-oval implants had strains concentrated only at the maxima; the minima (gaps) had no strain (Figure 2E).

We correlated these strain distributions with biological sequelae. Surrounding round implants was a ~150 µm circumferential zone in which no viable DAPI^+ve^ osteocytes were detectable (white arrow, Figure 2F). Most dying TUNEL^+ve^ osteocytes were found within this same zone (Figure 2F’. Around tri-oval implants, the tri-oval maxima had a similar distribution of dead and dying cells, but in the minima, viable osteocytes were abundant (Figure 2G; quantified in 2H). Dying osteocytes were significantly lower (Figure 2G’; quantified in I). The distribution of DAPI^+ve^ versus dead and TUNEL^+ve^ osteocytes was calculated (Figure 2H, I and Supplemental Figure S1); these data demonstrated that bone viability in the tri-oval minima—which comprised approximately 50% of the circumference of the implant—was significantly higher around the round implants.

### 3.3. Tri-oval Implants Exhibit Less Bone Resorption, which Allows them to Maintain their Stability Over Time

**Figure 3:**
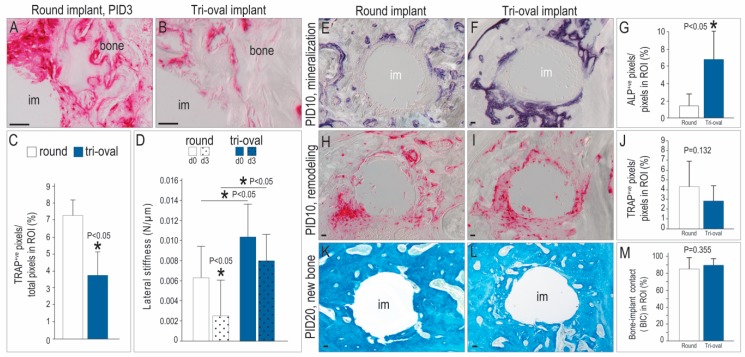
Tri-oval implants exhibits less bone resorption but more robust mineralization. (**A**) TRAP staining of interfacial tissues surrounding a representative round implant on PID3. (**B**) TRAP staining of the minima region around a tri-oval implant on PID3. (**C**) TRAP staining was quantified around the entire circumference of round and tri-oval implants. (**D**) Lateral stiffness test of round and tri-oval implants on PID0 and 3. (**E**) ALP staining of interfacial tissues surrounding a representative round and (**F**) a tri-oval implant on PID10, quantified in (**G**). (**H**) TRAP staining of interfacial tissues surrounding a representative round and (**I**) a tri-oval implant on PID10, quantified in (**J**). (**K**) Aniline blue staining of interfacial tissues surrounding a representative round and (**L**) a tri-oval implant on PID20; quantified in (**M**). Abbreviations as previously stated. Scale bars = 50 µm.

Peri-implant TRAP activity was more abundant around the round implants (Figure 3A) compared to the tri-oval implants (Figure 3B; quantified in Figure 3C). Resorption removes mineralized matrix, which reduces the elastic modulus of bone and leads to implant instability (white bars, Figure 3D). The tri-oval implants showed no significant loss in stability (blue bars, Figure 3D). Therefore, minimal TRAP activity observed around the tri-oval implants correlated with their greater stability after 3 days.

Eventually, both round and tri-oval implants showed evidence of new peri-implant bone mineralization (Figure 3E,F), although the amount of ALP activity was significantly greater around the tri-oval implants (quantified in Figure 3G). This new bone underwent normal remodeling (Figure 3H,I; quantified in Figure 3J). By PID20, both the round and tri-oval implants were fully surrounded by bone (Figure 3K,L; quantified in Figure 3M).

### 3.4. Tri-Oval Implants Exhibit Superior Osseointegration Compared to Conventional Round Implants

**Figure 4:**
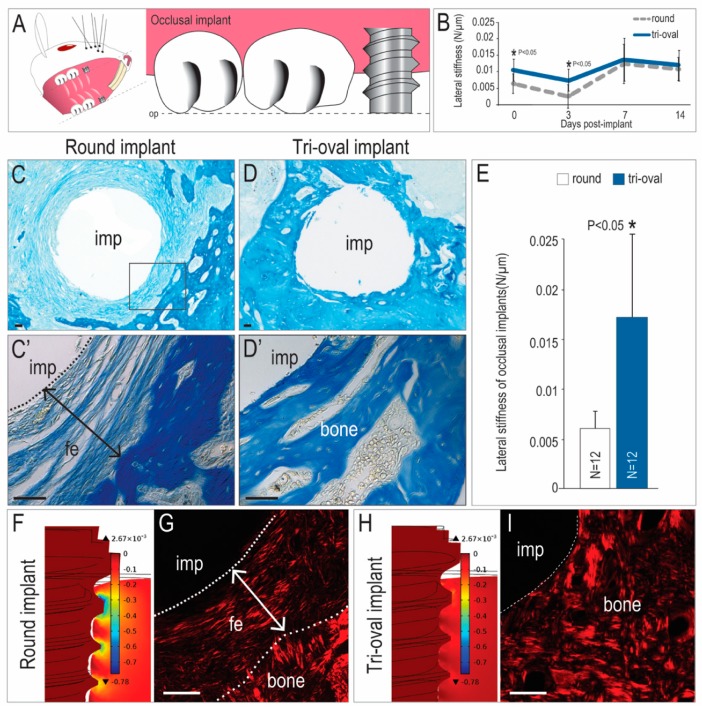
Stability over time as the function of implant geometry. (**A**) Schematic of an occlusal, or functional implant. (**B**) Quantification of lateral stability of sub-occlusal round and tri-oval implants at different timepoints. Aniline blue-stained tissue sections from PID20 through an (**C**,**C**’) occlusal round implant and (**D**,**D**’) an occlusal tri-oval implant. (**E**) Quantification of lateral stability of occlusal round and tri-oval implants on PID20. (**F**) In round occlusal implants, FE modeling of peri-implant strain on PID3 and (**G**) picrosirius-red stained tissues from PID20. (**H**) In tri-oval occlusal implants, FE modeling of peri-implant strain on PID3 and (**I**) picrosirius red-stained tissues from PID20. Abbreviations: op, occlusal plane; imp, implant; fe, fibrous encapsulation. Scale bars = 50 µm.

In the experiments conducted thus far, tri-oval implants exhibited better primary stability than round implants, yet both eventually were surrounded by bone. This result was not unexpected because in both cases, implants were placed sub-occlusally, and in previous studies we have shown that sub-occlusal round implants osseointegrate efficiently and effectively [31,32]. Moreover, no differences in quantity of bone or in lateral stability were detected at PID14 (Figure 4B).

We wondered if the fact that significantly better primary stability exhibited by the tri-oval implant would have a long-term benefit if the implants were immediately loaded. Both the round and tri-oval implants were subjected to functional loading, immediately after placement, which was achieved by positioning the very top of the implant at the same height as the adjacent molar (Figure 4A). The difference in outcome was dramatic: whereas the round implants underwent fibrous encapsulation (Figure 4C,C’), these tri-oval implants osseointegrated (Figure 4D,D’).

Lateral stability results were consistent with histologic/histomorphometric analyses: the soft interfacial tissues surrounding the round implant cases offered little support and consequently, the round implants exhibited poor secondary stability (i.e., small values of lateral stiffness). In comparison, the stiffer interfacial tissues around the tri-oval implants translated into larger lateral stiffness and thus higher secondary stability (Figure 4E).

### 3.5. The Magnitude of Interfacial Strain is a Key Influence on Whether an Implant will Undergo Fibrous Encapsulation or Osseointegration

Why did these round occlusal implants fail? The key to answering this question lies in the observation that the same round implants can osseointegrate, provided they are placed sub-occlusally to reduce loading (Figure 3). Thus, the round implants failed because they lacked sufficient primary stability (Figure 1Q). We sought to link this observation about stiffness at PID0 with the fates of the implants on PID20, and to do so, we turned again to FE modeling.

Implant stability is a function of the elastic modulus of peri-implant tissue; in other words, the stiffer the tissue, the less the implant will move under loading. FE modeling was used to back-calculate the peri-implant bone modulus that corresponded to the experimentally-measured lateral stability (see Materials and methods). At PID0 and PID3, the peri-implant tissues surrounding trioval implants were 3.5 times stiffer than those surrounding round implants. Using these modulus values, FE models demonstrated that peri-implant strains at PID0 and PID3 were significantly higher around the round occlusal implant (Figure 4F). For example, at the crestal thread tips of an occlusally-loaded round implant, principal compressive strain magnitudes reached >50% (Figure 4F). On the other hand, identically-loaded tri-oval implants were surrounded by stiffer peri-implant tissue and the resulting strains at PID0 and PID3 were less than 10% (Figure 4H). Collectively, these data provide critical insights as to why a round implant with significantly less primary stability underwent fibrous encapsulation when subjected to immediate loading (Figure 4G), whereas a tri-oval implant, with statistically higher primary stability, underwent osseointegration when subjected to the same immediate loading conditions (Figure 4I).

## 4. Discussion

We coupled mechanical testing with computational studies and histologic/immunohistologic analyses to assess how altering an implant’s geometry affected its ability to osseointegrate. We tested implants that were placed below the level of the occlusal plane, and those placed in function. In both scenarios, the tri-oval implants out-performed the round implants. Evidence supporting this conclusion came from mechanical, computational, and biological analyses.

### 4.1. The Maxima of a Tri-Oval Implant aid in Mechanical Stability

Compared to round implants, the tri-oval implants exhibited better primary stability, which was achieved without using a higher IT (Figure 1). The larger stability was achieved because the maxima of the tri-oval implant penetrated a greater distance into bone than did the threads of the round implant (Figure 2). Based on our data, one might legitimately ask if the novel tri-oval implant design would be negated simply by undersizing the osteotomy for the round implant. In this thought experiment, the threads of the round implant would penetrate deeper into bone and as a result the implant would presumably demonstrate better initial stability. But just as reliably, this scenario would also increase IT [8], peri-implant strain [11], and its associated micro-damage [8,15,33]. In turn, this micro-damage would increase the spatial extent of peri-implant bone resorption (Figure 3) during the early post-operative stages of bone remodeling, which would lower the net modulus of the peri-implant bone and result in a transient decrease of initial stability–as seen for example at PID 3 (Figure 4B).

Clinical observations are consistent with this line of reasoning: in multiple studies, sub-occlusal implants showed a decline in mean ISQ values between weeks 1-4 [34,35,36]. Friberg also reported a decrease in stability for a majority of sub-occlusal implants [37,38]. Our preclinical study appears to be the first to provide direct molecular, cellular, histologic, and mechanical data to explain how this transient “dip” in implant stability actually occurs.

### 4.2. The Minima of a Tri-Oval Implant Create a Pro-Osteogenic Environment

Fifty percent of the peri-tri-oval implant environment had very low/no strain (Figure 2E), where damage to the mineralized matrix is minimized, osteocyte death is minimal, and bone resorption is reduced [8,15,33]. Together these events culminated in significantly more new bone around the tri-oval implants (Figure 2 and Figure 3). A similar finding has been reported using a canine implant model, where investigators demonstrated that new woven bone forms first in regions where there is a gap in the bone-implant contact [12]. We find that areas of low/no strain strongly support osteoblast differentiation and new mineralized matrix deposition, provided the osteogenic potential of the bone is good [22].

### 4.3. Clinical Implications of this Study

Round-shaped implants can osseointegrate, even when subjected to loading immediately after placement. Why, then, did we observe that round implants failed to undergo osseointegration? The answer is straightforward: in those cases where round implants became encapsulated in fibrous tissue it was because loading was allowed on an implant that lacked sufficient primary stability (Figure 1). If the same implant—with the same degree of instability—was buried, then by PID20 it was surrounded by new bone (Figure 3). These data indicate the importance of an “unloaded” healing period proposed by Branemark [39]. What if the healing period is eliminated? Our data predict that healing periods between implant placement and loading could be shortened- or eliminated—without jeopardizing long-term implant success if osteocyte death was minimized during site preparation, and the implant had a geometry that provided both mechanical stability and a pro-osteogenic environment.

## 5. Conclusions

These multiscale biomechanical analyses demonstrated that the novel tri-oval implant design provided mechanically and biologically favorable environment for peri-implant bone formation and promoted osseointegration.

## Supplementary Materials

The following are available online at https://www.mdpi.com/2077-0383/8/4/427/s1, Figure S1. Method to determine the distribution of apoptotic osteocytes. (**A**) Using differential interference contrast (DIC), the peri-implant environment of round implants was visualized. (**B**) DAPI staining identified viable osteocytes in four zones circumscribing the implant. (**C**) Co-staining with TUNEL identified apoptotic osteocytes in four zones circumscribing the implant. (**D**–**F**) The same procedure was used to analyze the minima regions of tri-oval implants. Abbreviations: imp, implant. Scale bars = 50 µm.; Table S1. Osteotomy and implant parameters; Table S2. Osteotomy and implant parameters.
