# Supplementary material for: Mechanical and Biological Advantages of a Tri-Oval Implant Design
Source: J Clin Med. 2019 Mar 28;8(4):427. doi: 10.3390/jcm8040427 (PMC6517945; doi:10.3390/jcm8040427)
Supplement: Supplementary file 1 [file jcm-08-00427-s001.pdf]

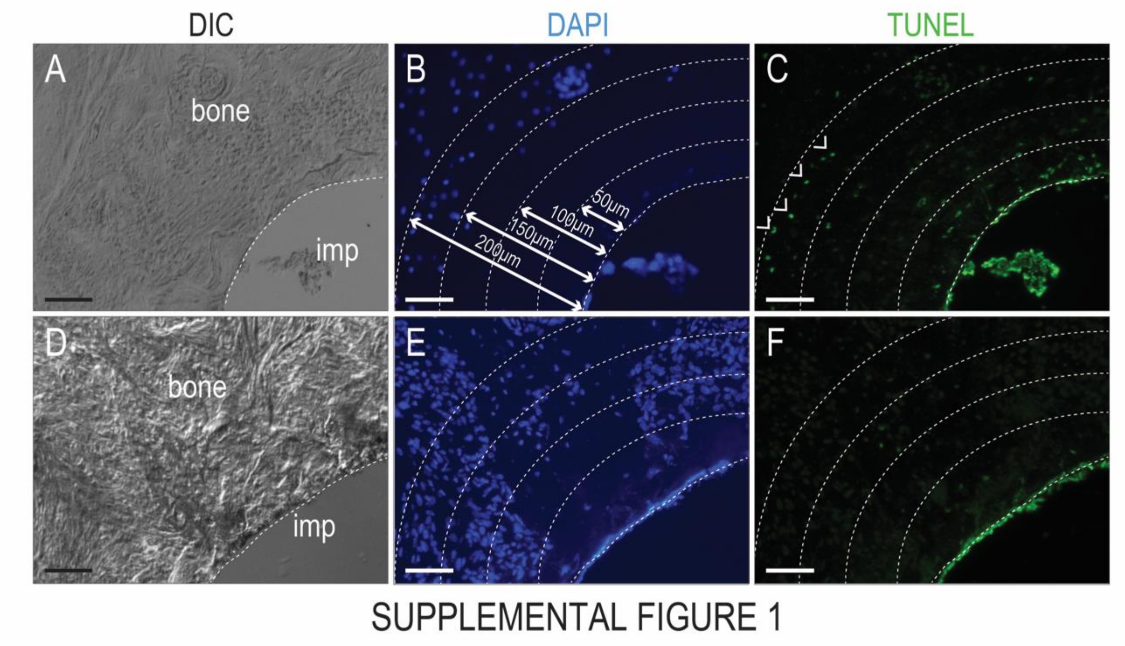

Supplemental Figure S1. Method to determine the distribution of apoptotic osteocytes**.** (**A**) Using differential interference contrast (DIC), the peri-implant environment of round implants was visualized. (**B**) DAPI staining identified viable osteocytes in 4 zones circumscribing the implant. (**C**) Co-staining with TUNEL identified apoptotic osteocytes in 4 zones circumscribing the implant. (**D**–**F**) The same procedure was used to analyze the minima regions of tri-oval implants. Abbreviations: imp, implant. Scale bars = 50μm.

Supplemental Table S1. Osteotomy and implant parameters.

Parameter	Osteotomy diameter (mm)	Round implant (mm)	Tri-oval implant (mm)
External diameter (mm)	0.45	0.5	0.5mean (0.54max, 0.42min),
Thread core diameter (mm)	-	0.4	0.44max, 0.36min
Thread pitch (mm)	-	0.15	0.15
Thread length (mm)	-	2	2
Misfit based on radius (mm)	-	0.025	0.045max, -0.015min

Supplemental Table 2. Experimental groups.

Experimental variables tested		Time points	# of animals	# of round implants	# of tri-oval implants
Micro CT, Histology		PED0	3	-	-
		PED28	3	-	-
Insertion torque		PID0	6	6	6
Lateral stability	Sub-occlusal	PID0	6	6	6
		PID3	6	6	6

testing		PID7	6	6	6
		PID14	6	6	6
		PID20	6	6	6
	Occlusal	PID0	6	6	6
		PID20	12	12	12
Histology, TUNEL, ALP/TRAP, IHC	Sub-occlusal	PID3	6	6	6
		PID7	6	6	6
		PID10	6	6	6
		PID20	6	6	6
	Occlusal	PID0	6	6	6
		PID20	6	6	6
	Subtotal		96	90	90

Abbreviations: PED, post-extraction day; PID, post-implant day; ALP, alkaline phosphatase; TRAP, tartrate-resistant acid phosphatase; IHC, immunohistochemistry.
